# Supplementary material for: Development of transgenic models susceptible and resistant to SARS-CoV-2 infection in FVB background mice
Source: PLoS One. 2022 Jul 26;17(7):e0272019. doi: 10.1371/journal.pone.0272019 (PMC9321403; doi:10.1371/journal.pone.0272019)
Supplement: S1 Raw images — (PDF) [file pone.0272019.s001.pdf]

S1_raw-images

Development of transgenic models susceptible and resistant to SARS-CoV-2 infection in FVB background mice

Sun-Min Seo[1], Jae Hyung Son[2], Ji-Hun Lee[1], Na-Won Kim[1], Eun-Seon Yoo[1], Ah-Reum Kang[1], Ji Yun Jang[2,3], Da In On[4], Hyun Ah Noh[4], Jun-Won Yun[5,6], Jun Won Park[7], Kang-Seuk Choi[8], Ho-Young Lee[9], Jeon-Soo Shin[10], Jun-Young Seo[11], Ki Taek Nam[11], Ho Lee[2*], Je Kyung Seong[12*], Yang-Kyu Choi[1*]

[1] Department of Laboratory Animal Medicine, College of Veterinary Medicine, Konkuk University, Seoul, Republic of Korea

[2] Graduate School of Cancer Science and Policy, National Cancer Center, Goyang, Gyeonggi, Republic of Korea

[3] College of Pharmacy, Dongguk University, Seoul, Republic of Korea

[4] Korea Mouse Phenotyping Center (KMPC), Seoul National University, Seoul, Republic of Korea

[5] Department of Medical and Biological Sciences, The Catholic University of Korea, Bucheon, Republic of Korea

[6] Laboratory of Veterinary Toxicology, College of Veterinary Medicine, Seoul National University, Seoul, Republic of Korea

[7] Division of Biomedical Convergence, College of Biomedical Science, Kangwon National University, Chuncheon, Republic of Korea

[8] Laboratory of Avian Diseases, College of Veterinary Medicine, Seoul National University, Seoul, South Korea

[9] Department of Nuclear Medicine, Seoul National University Bundang Hospital, Seongnam, Republic of Korea

[10] Department of Microbiology, Yonsei University College of Medicine, Seoul, Republic of Korea

[11] Severance Biomedical Science Institute, Brain Korea 21 Project for Medical Science, Yonsei University College of Medicine, Seoul, Republic of Korea

[12] Laboratory of Developmental Biology and Genomics, Research Institute for Veterinary Science, and BK 21 PLUS Program for Creative Veterinary Science Research, College of Veterinary Medicine, Seoul National University, Seoul, Republic of Korea

* ho25lee@ncc.re.kr (HL); * snumouse@snu.ac.kr (JKS); yangkyc@konkuk.ac.kr (YKC)

* HL, JKS and YKC are contributed equally to this work as co-corresponding authors.

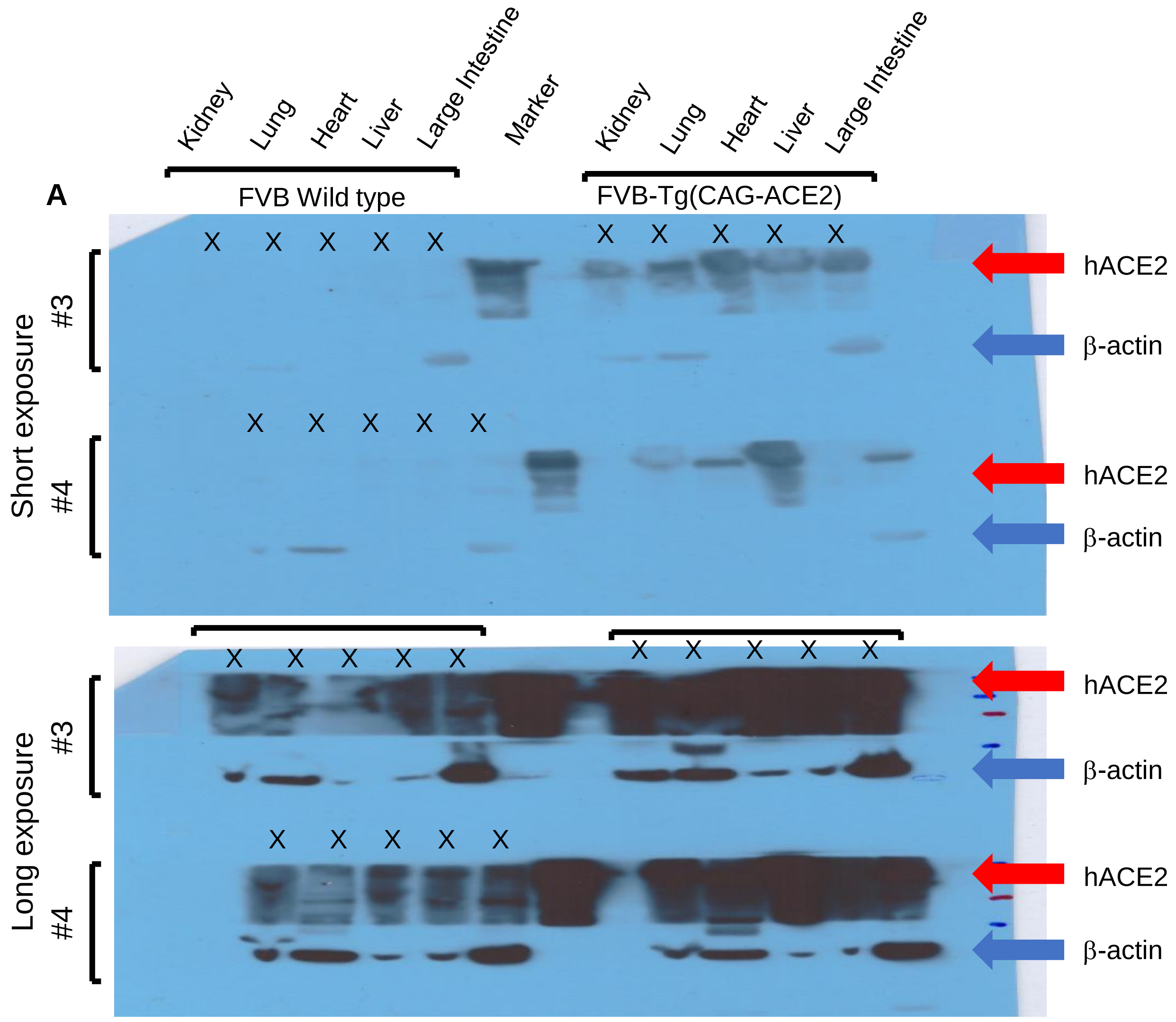

Uncropped images of blot presented in Fig 1A

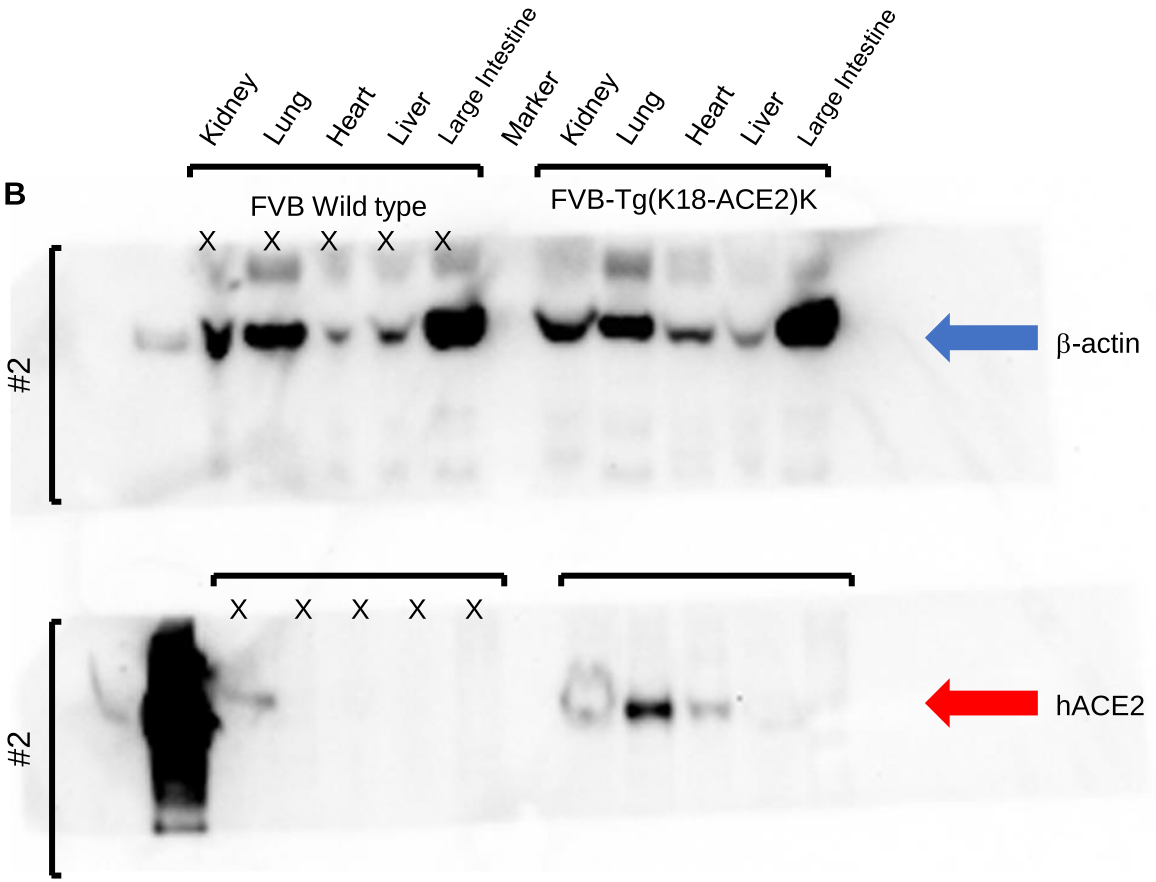

Uncropped images of blot presented in Fig 1B
