# Supplementary material for: Development of transgenic models susceptible and resistant to SARS-CoV-2 infection in FVB background mice
Source: PLoS One. 2022 Jul 26;17(7):e0272019. doi: 10.1371/journal.pone.0272019 (PMC9321403; doi:10.1371/journal.pone.0272019)
Supplement: Attachment — Submitted filename: Response to Reviewers_220704.docx [file pone.0272019.s003.docx]

Kui Li, Ph.D

Academic Editor

PLOS ONE

Dear Dr. Li

Subject: Development of transgenic models susceptible and resistant to SARS-CoV-2 infection in FVB background mice [PONE-D-22-06945R1]

Thank you for inviting us to submit a revised draft of our manuscript entitled, "Development of transgenic models susceptible and resistant to SARS-CoV-2 infection in FVB background mice" to PLOS ONE. We also appreciate the time and effort you and each of the reviewers have dedicated to providing insightful feedback on ways to strengthen our paper. Thus, it is with great pleasure that we resubmit our article for further consideration. We have incorporated changes that reflect the detailed suggestions you have graciously provided. We also hope that our edits and the responses we provide below satisfactorily address all the issues and concerns you and the reviewers have noted.

**Proposals from Academic Editor**

Thank you for submitting your manuscript to PLOS ONE. After careful consideration, we feel that it has merit but does not fully meet PLOS ONE’s publication criteria as it currently stands. Therefore, we invite you to submit a revised version of the manuscript that addresses the points raised during the review process.

While the manuscript is substantially improved, some minor issues remain. As the reviewers pointed out, some corrections and additional clarifications are needed

1. Please review your reference list to ensure that it is complete and correct. If you have cited papers that have been retracted, please include the rationale for doing so in the manuscript text or remove these references and replace them with relevant current references. Any changes to the reference list should be mentioned in the rebuttal letter that accompanies your revised manuscript. If you need to cite a retracted article, indicate the article’s retracted status in the References list and also include a citation and full reference for the retraction notice.

**Answer:** We revised our references based on “Vancouver” style in (p. 20, lines 497, 504, 507, and 509), (p. 21, lines 519, and 537), (p. 22, lines 545, 547, 549-550, 556, 558, and 564), (p. 23, lines 571), and (p. 24, line 599). We marked the revised sentences with red color.

2. While the revised manuscript is substantially improved, some minor issues remain. As you see from the reviewers' comments, some corrections and additional clarifications are needed.

**Answer:** We revised our manuscript based on reviewers’ comments.

**Review comments from Reviewer #1:**

1. Overall, the authors properly addressed the reviewers’ comments. But oddly, the authors revised figure 1 and switched the labeling of Fig1a and 1b. The revised fig.1 indicated that CAG-hACE2 mice organs (kidney, lung, heart, intestine) expressed high levels of hACE2, whereas the lungs of the k18-hACE2 mice were the primary organ expressing hACE2. This contradicts the rest of the data (fig3, 7, 8) and statement (lines 265-276) as well as previous studies. Please examine.

**Answer:** Thank you for providing these insights. In our original manuscript, there was an error in the labeling of Fig1a and 1b. So, we switched the labeling in the revised manuscript. In contrast to qPCR and histopathology performed in the small intestine, Western blot was performed in the large intestine. For this reason, it is considered that a contradiction appeared between the expression level of hACE2 and pathological scoring. We revised our manuscript (p. 8, line 190, marked with red color.), Fig 1. (from intestine to large intestine), and supplemental figure 1. (from intestine to large intestine).

**Review comments from Reviewer #2:**

The authors have made significant revision and addressed majority of the concerns. There are several minor errors that need to be corrected. Comments are listed below.

1. At several instances where a new text is added, the authors mention " a significant difference was observed" or used similar language throughout the text. This should be corrected to clearly mention what differences were observed, and whether the difference was increased or decreased between the groups. Also- include the significance of these differences.

**Answer:** Thank you for your suggestion. We have reflected this comment by (p. 10, lines 226-227), (p. 10, lines 233-234), (p. 11, lines 256-257), (p. 11, lines 262-263), (p. 11, lines 266-268), (p. 12, lines 291-293), (p. 13, lines 302-304), (p. 14, lines 328-329), (p. 14, lines 342-343), and (p. 14, lines 352-354). Revised and added sentences marked with red color.

2. The authors use the word " virus propagation" in certain tissue on numerous occasions. This should be corrected to virus titers or virus replication or virus load.

**Answer:** We agree with your assessment. We revised the word “virus propagation” to “viral replication” and marked it with red color in (p. 11, line 258, 264, and 268), and (p.17, line 416).

3. In the earlier iteration, this reviewer asked authors to provide justification for recommending use of K18-hACE2 mice for vaccine evaluation versus CAG-hACE2 mice for therapeutic assessment. However, this statement is still not well justified. Therefore, should be removed from the text or provide better explanation.

**Answer:** We agree that our conclusions lack proper explanation. So, we have clarified our conclusion with a revised sentence (p. 2, lines 46-47) (p. 18, lines 457-460) and marked it with red color.

Again, thank you for giving us the opportunity to strengthen our manuscript with your valuable comments and queries. We have worked hard to incorporate your feedback and hope that these revisions persuade you to accept our submission.

Sincerely yours,

Yang-Kyu Choi, D.V.M., Ph.D.

Department of Laboratory Animal Medicine,

College of Veterinary Medicine, Konkuk University,

120 Neungdong-ro, Gwangjin-gu, Seoul 05029, Republic of Korea

Tel: +82-2-2049-6113/Fax: +82-2-450-3037

E-mail: [yangkyuc@konkuk.ac.kr](mailto:yangkyuc@konkuk.ac.kr)
